# Retraction: “Designing Positive Psychology Interventions for Social Media: Cross-Sectional Web-Based Experiment With Young Adults With Cancer”

**DOI:** 10.2196/82724

**Published:** 2025-09-05

**Authors:** JMIR Editorial Office

**Affiliations:** 1 JMIR Publications, 130 Queens Quay E, Suite 1100, Toronto, ON, M5A 0P6, Canada, 1 416 583 2040

Following a request by the authors, the JMIR Publications Editorial Office is retracting the article “Designing Positive Psychology Interventions for Social Media: Cross-Sectional Web-Based Experiment With Young Adults With Cancer” by Lazard et al [1] due to concerns regarding the integrity and reliability of the survey data collected by an external contractor, which underpinned this article. As a result, the JMIR Publications Editorial Office, the editor in chief of the journal, and the authors have lost confidence in the results and conclusions of the published article.

## Author Statement

Lazard et al [1] used an external contractor for data collection in December 2021. In June 2025, the authors became aware of legal concerns about alleged fraudulent practices by the external contractor used to collect the survey data the article wholly relied on. The authors can no longer confidently verify how these data were collected nor confirm their legitimacy. Consequently, the authors believe that retraction of the article is the most responsible course to maintain the integrity of the scientific record due to a loss of confidence in the findings.

All authors agreed with the retraction of the article.
